# Differentiation of sea buckthorn syrups processed by high pressure, pulsed electric fields, ohmic heating, and thermal pasteurization based on quality evaluation and chemical fingerprinting

**DOI:** 10.3389/fnut.2023.912824

**Published:** 2023-02-14

**Authors:** Robert Sevenich, Maximilian Gratz, Beverly Hradecka, Thomas Fauster, Thomas Teufl, Felix Schottroff, Lucie Souckova Chytilova, Kamila Hurkova, Monika Tomaniova, Jana Hajslova, Cornelia Rauh, Henry Jaeger

**Affiliations:** ^1^Department of Food Biotechnology and Food Process Engineering, Technische Universität Berlin (TU Berlin), Berlin, Germany; ^2^Leibniz Institute for Agricultural Engineering and Bioeconomy (ATB), Potsdam, Germany; ^3^Institute of Food Technology, University of Natural Resources and Life Sciences (BOKU), Vienna, Austria; ^4^Department of Food Analysis and Nutrition, University of Chemistry and Technology (UCT), Prague, Czechia; ^5^BOKU Core Facility Food and Bio Processing, University of Natural Resources and Life Sciences, Vienna, Austria

**Keywords:** innovative food technologies, sea buckthorn, untargeted chemical fingerprinting, indicator compound, food quality

## Abstract

**Introduction:**

Impact of processing on product characteristics, sustainability, traceability, authenticity, and public health along the food chain becomes more and more important not only to the producer but also to the customer and the trust of a consumer toward a brand. In recent years, the number of juices and smoothies containing so called super foods or fruits, which have been “gently pasteurized,” has increased significantly. However, the term “gentle pasteurization” related to the application of emerging preservation technologies such as pulsed electric fields (PEF), high pressure processing (HPP) or ohmic heating (OH) is not clearly defined.

**Methods:**

Therefore, the presented study investigated the influence of PEF, HPP, OH, and thermal treatment on quality characteristics and microbial safety of sea buckthorn syrup. Syrups from two different varieties were investigated under the following conditions HPP (600 MPa 4–8 min), OH (83°C and 90°C), PEF (29.5 kV/cm, 6 μs, 100 Hz), and thermal (88°C, hot filling). Analyses to test the influence on quality parameters like ascorbic acid (AA), flavonoids, carotenoids, tocopherols, antioxidant activity; metabolomical/chemical profiling (fingerprinting) *via U-HPLC-HRMS/MS* (here especially flavonoids and fatty acids); sensory evaluation, as well as microbial stability including storage, were conducted.

**Results and discussion:**

Independent from the treatment, the samples were stable over 8 weeks of storage at 4°C. The influence on the nutrient content [Ascorbic acid (AA), total antioxidant activity (TAA), total phenolic compounds (TPC), tocopherols (Vit E)] was similar for all tested technologies. Employing statistical evaluation Principal Component Analysis (PCA) a clear clustering based on the processing technologies was observed. Flavonoids as well as fatty acids were significantly impacted by the type of used preservation technology. This was obvious during the storage time of PEF and HPP syrups, where enzyme activity was still active. The color as well as taste of the syrups were found to be more fresh-like for the HPP treated samples.

## 1. Introduction

During the last decades, consumer expectations of food and beverage products have changed considerably (1, 2) and “functional foods” as well as “superfoods,” i.e., products with special constituents or high levels of particular health benefitting and bioactive nutrients, became increasingly popular (2–5). Moreover, the demand for convenience and gentle processed food is still growing (6–9).

A product group that has the potential to meet the demand to be healthy and convenient at the same time, are fruit juices (10, 11), as especially fresh, cold-pressed juices, are considered to be a good source of bioactive compounds like vitamins and antioxidants (10, 12).

In this context, sea buckthorn became a promising product for consumers and producers, due to its natural high nutritive value. *Hippophae* is a genus of sea buckthorn, characterized by orange-yellow berries, which have been used over the centuries as food and traditional medicine, as well as skin treatment (13–16). *Leikora* (*Hippophae rhamnoides*) is a female cultivar which was bred in East Germany in the late 1970s (17). It produces an abundant large fruit, with a high content of ascorbic acid (1,200–1,400 mg/100 g). Compared to other cultivars, *Leikora* is more acidic and can be harvested in late September to early October of the year. *Hippophae Botanica* (sea buckthorn *Botanica*) is of eastern European origin, contains less acidic components and tastes much milder than other cultivars such as *Leikora*. Compared to *Leikora*, the content of ascorbic acid (∼610–700 mg/100 g) is minimally lower, but *Botanica* has a higher oil content. Here, harvesting is carried out in early August (13). Sea buckthorn berries and their products were reported to have a positive effect on human health, mainly due to their high content of antioxidants, both water- and fat- soluble, such as ascorbic acid, flavonoids, phenolic acid, tocopherols, and carotenoids (18–21).

The highest levels of health-promoting compounds and the highest retention of organoleptic properties can be expected for fresh cold-pressed juices. However, even for high acidic juices (such as sea buckthorn), stored under cold conditions, only limited shelf life and microbial safety can be expected (11). Therefore, they are subjected to pasteurization aiming to eliminate microorganisms (bacteria, yeast, molds, and their thermal resistant spores) and inactivate enzymes that may adversely impact the flavor and total appearance. However, pasteurization effect can be enhanced by adding sugar (sucrose), supporting reduction of water activity. The reduction of sour taste of sea buckthorn by addition of sugar, transforming juice into syrup, improve the palatability (22). The traditional preservation techniques, such as thermal pasteurization, typically causes at least partial degradation of the nutritive, organoleptic, or physiochemical properties of the final product. To maintain valuable components and, at the same time, ensure the microbial safety, so-called emerging or alternative technologies have been investigated and partly applied in the food industry (23). These technologies include high pressure processing (HPP), pulsed electric field (PEF), or ohmic heating (OH) treatment (24, 25).

High pressure processing (HPP) is a non-thermal pasteurization technology where the juice inside the final package is subjected to high pressure (300–800 MPa) for a few minutes (< 10 min). The technology allows gentle pasteurization at room temperature (24, 26, 27). PEF is another non-thermal preservation technology that involves the application of short (μs-ms range) high electric field pulses (> 10 kV/cm) to the liquid. Furthermore, PEF allows pasteurization at lower temperatures compared to a conventional thermal treatment, although the temperature increases due to the applied electrical current (25, 28). This effect is used for OH treatment where an electrical current with a lower field strength (< 1 kV/cm) is applied, with the aim of rapidly and uniformly heating the product (29, 30). All three technologies are already commercialized and several studies over the past two decades could show the potential of these emerging technologies to provide fresh like fruit juices or syrups with a high nutritional quality retention and an extended shelf life (12, 31–34).

In order to provide a basis for appropriate labeling and consumer transparency, indicators and standard analyses need to be established to differentiate the various processing technologies. A well-suitable tool in this context could be non-targeted fingerprinting analyses (35). Therefore, this paper presents a comprehensive study of the impact of pulsed electric field (PEF), ohmic heating (OH), high pressure processing (HPP), and conventional thermal pasteurization on the quality and safety of two types of sea buckthorn syrups during a storage period of 2 months. The potential of emerging technologies to maintain the perceived freshness, organoleptic, and nutritional quality of the syrups was analyzed using various chemical and sensorial analyses. Microbial safety was verified by employing microbial analyses. Furthermore, by using non-targeted fingerprinting, characteristic compounds and profiles for each processing technology were identified. The results of this study might help to find characteristic indicators that can be linked to specific processing technologies and therefore support future food authentication approaches.

## 2. Materials and methods

### 2.1. Berry samples and syrup production

Both buckthorn varieties (*Botanica and Leikora*) were provided by a small Czech organic orchard farmer, who processed the fresh berries prior to the subsequent juice and syrup making. Processing steps were carried out by the farmer as follows: (i) variety *Botanica*–berries were gently crushed with a speed mixer for 20 s and cooled; (ii) variety *Leikora*–berries were crushed similarly to *Botanica* berries and then soaked in water (1 L of berries and 0.3 L of water) for 24 h and placed in a dark, cool place. The berries were transported by the University of Chemistry and Technology Prague, to the Technical University of Berlin, and the University of Natural Resources and Life Sciences, Vienna. In total, an amount of 90 kg of sea buckthorn berries was used for the trials.

The crushed berries were pressed with a Philips HR1949/20 slow juicer and mixed. Due to the high acidity of sea buckthorn juice (containing relatively high content of organic acids), sugar was added to improve the sensory attributes. In case of the *Botanica* variety 0.5 kg of sugar to 1 L of juice was added and in case of *Leikora* variety 0.7 kg of sugar to 1 L of juice.

The blended syrup was then used for the experiments (Table 1). The final syrups were characterized by the following values: pH 2.7 and 2.8, electrical conductivity 0.87 and 1.2 ms/cm, 35°Brix (density 1,130 kg/m^3^) and 40°Brix (density 1,130 kg/m^3^) for the varieties *Leikora* and *Botanica*, respectively.

**TABLE 1:** Overview of the process parameters used for the two types of sea buckthorn syrups.

Process	Low intensity	High intensity
Inpack water bath heating (WB)	-	Tmax 89°C Time 2 min
Aseptic filling (AF)	T_max_ 80°C Time 15 s	T_max_ 87.5°C Time 15 s
Hot filling (HF + C, HF)	T_max_ 88°C Time 15 s Cooling 10°C	T_max_ 88°C Time 15 s No cooling
Ohmic heating (OH)	T_max_ 85°C Power 0.8 kW Time 15 s	T_max_ 91°C Power 0.9 kW Time 15 s
High pressure processing (HPP)	T_max_ 35°C Pressure 600 MPa Time 4 min	T_max_ 35°C Pressure 600 MPa Time 8 min
Pulsed electric fields (PEF)	E 29.5 kV/cm, f 100 Hz, τ 6 ms, mean residence time 12.1 ms, T_*in*_ 40°C, T_*out*_ 67°C, EI 111 kJ/kg^1^	-

Deviations in maximum temperature (T_max_) in the holding section of thermal treatments (AF, aseptic filling; OH, ohmic heating; HF, hot filling) were ± 0.5°C, during the HPP treatment ± 1°C. Further abbreviations refer to: WB (water bath), AF (aseptic filling), HF (hot filling without cooling), HF + C (hot filling with cooling), HPP (high pressure processing, 600 MPa), PEF (pulsed electric fields), and OH (ohmic heating). ^1^E refers to the electric field strength, f is the frequency of the applied electric field, τ the pulse width, and EI is the energy input.

### 2.2. Preservation and storage of syrups

Ready-made syrups (*Leikora* and *Botanica* varieties) were divided into six batches and subjected to the following pasteurization treatments: pulsed electric field (PEF), ohmic heating (OH), high pressure processing (HPP), as well as different thermal treatments. For the latter the following processes were selected to investigate their influence on the nutritional profile based on their different thermal load applied to the product. A continuous short time pasteurization treatment, using tubular heat exchanger combined with aseptic filling (AF), was used. Further, hot filling was carried out, which is a semi-continuous process involving a tubular heat exchanger and a filling unit, with subsequent cooling (HF + C) and without cooling (HF). This was done to investigate if longer treatment times, as well as in-pack cooling have an influence on the nutritional profile of the syrup. Lastly, water bath heating (WB) of the bottled syrup was accomplished, at a core temperature of 89°C for 2 min, to simulate a “worst case” heat treatment, such as the preparation of this product at home. For each of the chosen preservation technologies, a low- and high-intensity treatment was applied (see Table 1), in order to study different possible process windows–except for WB and PEF, which was due to technical limitations of the used equipment. For HPP and WB, 300 mL of the syrups were filled prior to processing in plastic bags made of polyethylene/polyamide (Luckfield and Mann GmbH, Kiel, Germany). For continuous pasteurization treatments (AF, OH, and PEF), the syrups were aseptically filled into 250 mL sterile glass bottles. In addition, the hot filling process (HF, HF + C) was performed using the same glass bottles.

Process parameters were chosen based on similar expected microbiological inactivation levels for the low and high intensity treatments, respectively. Therefore, parameters were derived from literature, but also taking into consideration common industrial processing regimes for syrup and juice pasteurization.

For this purpose, the tubular heat exchanger was selected as a standard conventional heating technology for industrial applications. Therefore, the other processing regimes were selected based on the parameters chosen for this production system. The standard industrial parameters were chosen to be 80°C for 15 s (low intensity), as well as 87.5°C for 15 s (high intensity), based on typical industrial use cases (36). Based on this, *P*_80°*C*_-values (*z*-value 8°C) were calculated and used as a base for comparison. Obtained values were 0.5 and 2.5 min for low and high intensity treatments, respectively. Next, Ohmic Heating was used as a common innovative thermal process. Therefore, the intensity levels were also matched to 0.5 and 2.5 min, respectively. Hot filling as well as water bath heating were chosen as “worst case” industrial processes commonly used by small and medium sized food companies, known to exert high thermal loads leading to overprocessing. Consequently, these technologies were characterized by higher *P*_80°*C*_-values compared to the two previously mentioned technologies. In this regard, low intensity hot filling (with cooling) was in the same range as the samples treated with the high intensity tubular heat exchanger (*P*_80°*C*_ of 2.5 min), whereas the high intensity treatments showed a distinctly greater value of 20 min. In terms of water bath heating, the treatment was again in the same range as the high intensity hot filling trials.

Considering the non-thermal treatments, *P*_80°*C*_ values were not used as a base of comparison, as the occurrence of additional inactivation effects leads to a distinctly lower thermal load. Therefore, typical industrial processing conditions were chosen for the HPP and PEF trials, leading to pasteurization conditions of 5 log_10_ inactivation (37–39) in order to obtain a microbially safe product.

Ultimately, the impact of these different industrial processing regimes on the product’s quality markers was studied, in order to evaluate if the different technologies can be differentiated by metabolic fingerprinting.

#### 2.2.1. Thermal treatments

##### 2.2.1.1. Conventional continuous pasteurization

A pilot-scale treatment system (HT220-DSI, OMVE, De Meern, Netherlands) was used to perform the aseptic filling and hot filling treatments. The system is driven by a progressive cavity pump and equipped with tubular heat exchangers for preheating, main heating, and cooling. After the main heating section, an insulated holding section is mounted with a residence time of 15 s. The outlet of the system was connected to a laminar flow cabinet by a sterile tube, for aseptic filling (see below). The volume flow was set to 20 L/h for all trials using this equipment.

##### 2.2.1.2. Pasteurization by ohmic heating

For continuous ohmic heating treatments, this equipment was combined with an OH system (German Institute of Food Technologies–DIL, Quakenbrück, Germany) consisting of an OH generator (12 kHz, bipolar rectangular pulses, peak voltage 1,000 V, pulse width ∼40 μs) and a co-linear treatment chamber (1 cm inner diameter, 4 heating zones with an electrode distance of 4.5 cm). This combination of tubular heat exchangers with the OH system enabled fast pre-heating before the OH treatment and fast cooling directly afterward. Power levels used for OH were in the range of 0.8–0.9 kW.

##### 2.2.1.3. Filling

After conventional pasteurization and OH, aseptic filling was carried out into sterile glass bottles (250 mL) within a vertical laminar airflow cabinet (Steril Gemini, Apeldoorn, The Netherlands). Hot filling was carried out after heating the syrup to a temperature of 88°C using the tubular heat exchanger, into the same glass bottles, with and without external cooling (cold water bath at 10°C for 30 min).

##### 2.2.1.4. Water bath heating

Furthermore, heating of filled glass bottles in a water bath (WB; set temperature: 95°C) was carried out until a core temperature of 89°C was reached. It took approximately 10 min until this temperature was reached. Afterward, the temperature was held constant for 2 min. The temperature during the heating was monitored using a thermocouple (Testo 925, Type K, Testo SE and Co., Titisee-Neustadt, Germany). The bottles were subsequently cooled in an ice bath.

#### 2.2.2. High-pressure processing

The U 4,000 high-pressure vessel (volume of 0.75 L, Unipress, Warsaw, Poland) was used for the high-pressure treatment of the sea buckthorn syrups (600 MPa, initial temperature: 35°C, holding times: 4 and 8 min). Process parameters were chosen based on the industrial standard as well as on expected microbial reduction based on scientific literature (37, 40, 41). Before treatment, the plastic bags (polyethylene and polyamide layer; Luckfield and Mann GmbH, Kiel, Germany) containing the syrup (300 mL) were vacuum sealed (Plus Vac 23, KOMET Maschinenfabrik GmbH, Plochingen, Germany) and stored at 8°C until processing. Prior to the treatment, the samples were placed in the pressure chamber and a mixture of water and 1,2-propanediol (1:1, *v/v;* Carl Roth GmbH and Co. KG, Karlsruhe, Germany) served as the pressure-transmitting medium. When 600 MPa were reached, the time measurement began. The temperature rise in the high-pressure vessel caused by the adiabatic heat of compression was max. 20°C, i.e., the final temperature of the syrup never exceeded 35°C. At the end of the holding time, the pressure was automatically released, and the bags were removed. The treated samples were cooled immediately.

#### 2.2.3. Pulsed electric fields

Pulsed electric fields (PEF) experiments were conducted using a continuous PEF equipment described in detail by Reineke et al. (42). Briefly, the first component was a laboratory-scale progressive cavity pump (Hanning Elektro-Werke GmbH and Co. KG, Oerlinghausen, Germany) operating at a mass flow rate of 14.8 kg/h. A stainless-steel tempering coil (Technical University of Berlin) was used to preheat the different syrups to the desired inlet temperatures (∼40°C). Downstream of the pre-heating zone, the syrups entered a co-linear treatment chamber, which is described in detail by Meneses et al. (43). Briefly, the PEF cell consisted of a cylindrical central high voltage electrode (stainless steel), surrounded by two cylindrical Teflon^®^ insulators and cylindrical top and bottom ground electrodes (stainless steel). For the trials, a 7 kW pulse modulator (ScandiNova Systems AB, Uppsala, Sweden) was used. PEF treatment parameters encompassed a pulse repetition frequency of 100 Hz, an electric field strength of 29.5 kV/cm, and a pulse width of 6 μs. Mean residence time of volume elements within the electric field was 12.1 ms. Treatment parameters were chosen based on the expected microbial inactivation in juices, as derived from literature (44, 45), as well as the possible specifications the used generator could provide. The outlet temperature was 67°C,

#### 2.2.4. Storage and sampling

An overview of the treated samples and the process parameters as well as the different processing intensities is given in Table 1. The treated samples were stored at 8°C and chemical, sensory, and microbial analyses were performed every 2 weeks for a maximum storage time of 8 weeks. All experiments were carried out as biological as well as process duplicates. The samples of AF, HF, HF + C, WB, and PEF were stored in glass bottles, since this was the packaging used during the respective trials. For the HPP treatment, flexible plastic bags consisting of one polyethylene and one polyamide layer were used, since cans or glass bottles would break during the treatment. The double layer serves as an oxygen barrier and therefore has similar protective properties as the glass bottles.

### 2.3. Microbiological analyses

The microbial load was determined for the untreated and treated syrups, as well as during the storage period, directly after the respective treatment and each storage period. Samples were serially diluted using sterile Ringer’s solution and 0.1 mL of each dilution was plated on appropriate agar. Standard I Nutrient Agar (Merck KGaA, Darmstadt, Germany) was used to determine the total aerobic mesophilic bacterial counts. The agar plates were incubated at 30°C for 72 h and the colony forming units were counted. Yeast-Malt Agar (Y 3127-500G, Merck KGaA, Darmstadt, Germany) was used for the cultivation of surviving yeasts as well as molds. Here, counting was carried out after incubation at 25°C for 96 h.

### 2.4. Targeted quality analyses

#### 2.4.1. Physicochemical parameters (pH, soluble solid content, color)

The pH was measured with a pH meter from Mettler Toledo (1120) and the soluble solid content with a digital refractometer (Krüss DR201-95). The L*, b*, and a* values were determined by using the DIGIEYE (VeriVide) non-contact digital imaging system. Based on these values, the color difference (ΔE) was calculated according to the following equation (Eq. 1) (46, 47). The L*, b*, and a* values were obtained by measuring each sample twice at different locations.


(1) $$\Delta \,E=\sqrt{\left(\Delta \,L^{2}+\Delta \,a^{2}+\Delta \,b^{2}\right)}$$

#### 2.4.2. Analysis of ascorbic acid

The sample (10 g) was diluted with 100 mL of metaphosphoric acid (3%, w/v) and analyzed by high performance liquid chromatography coupled to a diode array detector (HPLC-DAD) (Agilent Technologies 1200, USA). For chromatographic separation, a C18 column (125 × 4 mm, 5 μm) (Supelco, Germany) was used and the temperature was set at 35°C. Isocratic elution was used with a mobile phase consisting of methanol: water (5:95, *v/v*) and pH adjusted to 3 with phosphoric acid. The flow rate was set at 0.8 mL/min and sample injection 20 μL. Ascorbic acid was detected at 254 nm. Quantification was performed with and ascorbic acid standard applying an external calibration ranging from 0.1 to 100 mg/L. The method was validated with a determined uncertainty of 10%.

#### 2.4.3. Analysis of carotenoids and tocopherols

The analytical approach was similarly performed as described by Bhave et al. (48) with slight modifications including alkaline hydrolysis. Briefly, 8 mL of hexane and a 2 mL of a mixture of ethanol/acetone (6:4, *v/v*) containing 0.2% (*w/w*) of tert-butyl-hydroxytoluene (t-BHT) was added to a 1 g of sample and properly shaken for 5 min. The alkaline hydrolysis was performed for 18 h with potassium hydroxide in methanol (1:50, w/v). For neutralization sodium sulfate in deionized water (100 g/L, w/v) was added together with 10 mL of hexane and shaken for 2 min. The sample was then centrifuged at 10,000 *g* for 5 min. The upper hexane layer was collected into a 50 mL evaporative flask. The samples were reextracted three times with 10 mL of hexane until colorless. The collected hexane layers were then evaporated to dryness on a rotary shaker, reconstituted in a mixture of ethanol/acetone (6:4, *v/v*) containing 0.2% (*w/w*) of tert-butyl-hydroxytoluene (t-BHT) and filtered by a 0.2 mm PVDF (polyvinylidene fluoride) membrane filter. The mobile phase consisted of (A) 100% (v/v) acetonitrile and (B) 90% (v/v) acetonitrile. The elution gradient with constant flow was as follows 0.5 mL/min: 0–2 min (100% B), 5–28 min (100% A), and 28.5–30 min (100% B). The sample injection was 3 μL. For chromatographic separation, a Poroshell 120 EC-C18 column (2.1 × 100 mm, 2.7 μm) (Agilent Technologies, USA) was used. The column temperature was set at 30°C. Analytes were detected simultaneously by HPLC-DAD (Agilent Technologies 1200, USA) for the determination of carotenoids (lutein and zeaxanthin wavelength 444 nm and β- carotene 450 nm) and the fluorescence detector (FLD) for the determination of α-, β-, and γ- tocopherols at excitation and emission wavelengths 290 and 333 nm, respectively. The content of tocopherols was expressed as vitamin E activity.

#### 2.4.4. Antioxidant reducing capacity

The antioxidant reducing capacity of the syrups was determined spectrophotometrically on the basis of the Folin-Ciocalteu assay (reagent: mixture of phosphowolframic and posphomolybdenic acid) and expressed as gallic acid equivalents. For this purpose, a methanolic:water solution (75:25, *v/v*) of freshly prepared gallic acid was prepared, with a concentration ranging between 2 and 500 mg/L. About 1 g of the sample was diluted in 10 mL of deionized water (Milli-Q purification system) and for the measurements, 15 μL of sample and 165 μL of Folin-Ciocalteu assay (1:9, *v/v* in water) were placed on the microplate. Deionized water was used as a blank. After 3 min, 140 μL of sodium carbonate (9%, *w/w*) was added and the microplate was placed in a dark place for 60 min. Using a Spectrophotometer Epoch Microplate reader (BioTek Instruments, USA), the absorbance was measured at 765 nm. The method was validated with a determined uncertainty of 10%.

#### 2.4.5. DPPH radical scavenging capacity

Antioxidant activity was determined spectrophotometrically on the basis of the measurement of DPPH radical scavenging activity as described by Bhave et al. (48) with slight modifications. 1 g of sample was diluted with 10 mL deionized water (Milli-Q purification system; Millipore). To determine antioxidant activity, a methanolic solution containing DPPH (2,2-diphenyl-1-picrylhydrazyl) radicals (52 mg/100 mL) was used. To calculate the concentration of antioxidants, expressed as equivalents of ascorbic acid, an aqueous solution of freshly prepared L-ascorbic acid, with a concentration ranging between 1 and 20 mg/L was prepared. Deionized water was used as a blank. The reduction of DPPH free radicals to DPPH_2_ (diphenylpicrinehydrazine) indicated by a change in color from violet to yellow was measured after 60 min at 517 nm using a Spectrophotometer Epoch Microplate reader (BioTek Instruments, USA). The method was validated with a determined uncertainty of 10%.

#### 2.4.6. Non-target analysis

An ultra-high performance chromatograph (Dionex UltiMate 3000 RS U-HPLC system, Thermo Fisher Scientific, Waltham, MA, USA) coupled to quadrupole-time-of-flight high resolution mass spectrometer (SCIEX TripleTOF^®^ 6600, Concord, ON, Canada) was used for metabolomic fingerprinting of processed sea buckthorn syrups. Briefly, 1 g of sample was diluted with 5 mL of methanol and shaken for 30 min. The solution was then centrifuged for 5 min at 10,000 *g* and filtered using a 0.2 mm PVDF membrane filter. The analytical strategy used with U-HPLC-HRMS/MS was similar to that described by Hurkova et al. (49). For chromatographic separation an Acquity BEH C18 column (100 × 2.1 mm × 1.7 μm) (Waters, Milford, MA, USA) was used. The column temperature was set at 60°C and the autosampler at 5°C. The mobile phase (A) consisted of water:methanol (95:5, *v/v*) with 0.1% formic acid and 5 mM ammonium formate and (B) consisted of isopropanol:methanol:water (65:30:5, *v/v*) with 0.1% formic acid and 5 mM ammonium formate. The elution gradient with constant flow was as follows 0.4 ml/min: 0.0 min (90% A), 1.0 min (50% A), 5.0 min (20% A), 11.0 min (0% A), 19.0 min (0 A), 19.1 min (90% A), 21.0 min (90% A). The injection volume of the sample was 2 μL. The setup of the mass spectrometer was as follows: nebulizing gas pressure: 50 psi; drying gas pressure: 50 psi; capillary voltage: + 4,500 V (negative mode −4,000 V); temperature: 500°C (positive mode), 450°C (negative mode), and declustering potential: 80 V. The monitored mass range was from m/z 100 to 1,200 Da for the full scan experiment, and in parallel production ion (PI) spectra from *m/z* 50 to 1,200. The collision energy was set to 35 V with a collision energy spread of 15 V. Qualitative analysis was performed with PeakView software (version 2.2, SCIEX, Concord, ON, Canada) equipped with MasterView and FormulaFinder functions for the estimation of the molecular formula and structural elucidation. The identification and confirmation compounds were conducted by comparing the measured MS/MS spectra with those available in on-line databases such as, e.g., Metlin.^1^

### 2.5. Data processing and statistical analysis

MarkerView software (version 1.3, SCIEX, Concord, ON, Canada) was used for both data processing and statistical evaluation. Prior to multivariate analysis (Principal Component analysis, PCA), several steps for data processing, including data filtering, peak and mass alignment (tolerance 0.02 Da), exclusion of background ions and non-monoisotopic ions, were carried out. The peak alignment for retention time from 0 to 12 min was set at 0.2 min. The processed data representing a data matrix that included molecular features was characterized by retention time, *m/z* value, and peak intensity. Logarithmic transformation and Pareto scaling were used as a data pre-processing method prior to the actual PCA. The data matrix was then subjected to total area sum normalization.

### 2.6. Sensorial evaluation

Sensory evaluation was conducted according to the international standard ISO 8589, 2007 in a standard accredited sensory laboratory. Samples (diluted syrup with fresh water, 1:10, *v/v*) were served according to the international standard (ISO 6658, 2005) and evaluated by 13 trained panelists (International standard ISO 8586, 2015). The samples were tested in their fresh state (storage point 0) and after 8 weeks of storage time (storage point 8). These storage times were selected since storage point 0 represents the state of the product right after the treatment and storage point 8 represents the “best before” date for the majority of PEF or HPP treated products. The samples were evaluated applying hedonic testing with a 10-point evaluation scale including intensity of color, acid taste, sweet taste, sea buckthorn taste, after taste, pleasantness of acid taste, sweet taste, sea buckthorn taste, total taste. In addition, a sequence and preference tests were performed with sea buckthorn samples stored for 8 weeks. In the case of the sequence test, samples treated with PEF, HPP, OH, and WB were evaluated. The preference test was aimed to evaluate only the samples processed under mild technologies such PEF, HPP, and OH. In case of *Botanica* variety, the PEF treated sample was excluded from the preference test due to microbiological contamination.

## 3. Results and discussion

### 3.1. Microbiological inactivation

Many articles and reviews have reported the promising use of PEF, OH, and HPP for the inactivation of pathogenic microorganisms in foods to obtain the needed food safety (50–54). However, there are only few papers in which inactivation after treatment and possible recovery of microorganisms during storage in combination with changes in quality attributes *via* targeted and untargeted chemical analyses were assessed. Two recent publications from Yildiz et al. (55) and Wibowo et al. (33), who compared non-thermal technologies with thermal pasteurization for the treatment of strawberry juice respectively apple juice, showed to a certain extent similar results concerning the inactivation respectively highlighted the gentler treatment of the non-thermal technologies. Although not so much detail was put on secondary metabolites.

In the untreated samples, a mean of 0.3–0.4*10^2^ CFU/g was present for aerobic mesophilic count Table 2 in BO syrup and LK syrup. In the untreated syrups, microorganisms increased from 10^7^ to 10^8^ CFU/g in the BO syrup and up to 10^4^ CFU/g in the LK syrup during the storage period (6 and 8 weeks). This could be attributed to the slightly lower pH of the LK syrup (2.6 vs. 2.86). It was obvious that when HPP (600 MPa at 4 and 8 min), OH or another thermal treatment was applied, the microorganisms were inactivated, and no recovery was observed over the storage period. The samples treated with PEF presented a different result. Directly after the treatment, the reduction compared to the untreated samples was rather nominal. After 2 and 8 weeks, the microorganisms recovered and were able to grow in the samples.

**TABLE 2:** Aerobic mesophilic counts of the stored sea buckthorn syrups according to pasteurization treatment.

Variety	Treatment	Aerobic mesophilic count [CFU/mL]				
		0 Week	2 Weeks	4 Weeks	6 Weeks	8 Weeks
BO	Untreated	43 ± 8	5.83*10^4^ ± 1.98*10^3^	4.28*10^7^ ± 1.98*10^6^	1.54*10^7^ ± 6.36*10^5^	2.12 *10^8^ ± 1.48*10^7^
LK	Untreated	30 ± 6	2.67*10^4^ ± 0.89*10^3^	1.15*10^4^ ± 0.20*10^4^	5.15*10^4^ ± 1.20*10^4^	<10^1^
BO	HPP 4 min	<10^1^	<10^1^	<10^1^	<10^1^	<10^1^
LK	HPP 4 min	<10^1^	<10^1^	<10^1^	<10^1^	<10^1^
BO	HPP 8 min	<10^1^	<10^1^	<10^1^	<10^1^	<10^1^
LK	HPP 8 min	<10^1^	<10^1^	<10^1^	<10^1^	<10^1^
BO	PEF	10 ± 1	77 ± 28	-	-	115 ± 9
LK	PEF	5 ± 2	42 ± 7	-	-	10 ± 1
BO	OH 85°C	<10^1^	<10^1^	<10^1^	<10^1^	<10^1^
LK	OH 85°C	<10^1^	<10^1^	<10^1^	<10^1^	<10^1^
BO	OH 91°C	<10^1^	<10^1^	<10^1^	<10^1^	<10^1^
LK	OH 91°C	<10^1^	<10^1^	<10^1^	<10^1^	<10^1^
BO	HF 88°C	<10^1^	<10^1^	<10^1^	<10^1^	<10^1^
LK	HF 88°C	<10^1^	<10^1^	<10^1^	<10^1^	<10^1^
BO	HF + C 88°C	<10^1^	<10^1^	<10^1^	<10^1^	<10^1^
LK	HF + C 88°C	<10^1^	<10^1^	<10^1^	<10^1^	<10^1^
BO	AF 80°C	<10^1^	<10^1^	<10^1^	<10^1^	<10^1^
LK	AF 80°C	<10^1^	<10^1^	<10^1^	<10^1^	<10^1^
BO	AF 87°C	<10^1^	<10^1^	<10^1^	<10^1^	<10^1^
LK	AF 87°C	<10^1^	<10^1^	<10^1^	<10^1^	<10^1^
BO	WB 89°C	<10^1^	<10^1^	<10^1^	<10^1^	<10^1^
LK	WB 89°C	<10^1^	<10^1^	<10^1^	<10^1^	<10^1^

WB (water bath, 89°C), AF (aseptic filling, 80–87.5°C), HF (hot filling at 88°C without cooling), HF + C (hot filling at 88°C plus cooling at 10°C), HPP (high pressure processing, 600 MPa/35°C/4 and 8 min), PEF (pulsed electric fields, 29.5 kV/cm, 6 μs, 100 Hz, Inlet 40°C and outlet 67°C), OH (ohmic heating, 0.8–0.9 kW, 85–91°C); LK (*Leikora*) and BO (*Botanica*).

In general, the load of yeast and molds was higher compared to the aerobic count. In the untreated samples, a mean of 0.15–0.09*10^3^ CFU/g of yeast and molds was found (Table 3) in BO syrup and LK syrup. In the untreated syrups, the microorganisms grew over the storage period (6 and 8 weeks) to 10^5^ CFU/g for the BO and LK syrup. The storage time growth is 10^3^ times lower as could be seen in case of the aerobic count (Table 2). This can be explained by the low resistance of yeasts and molds to low pH. When HPP, OH, or thermal treatment was applied, the microorganisms were inactivated, and no recovery was observed over the storage period. Compared to the results obtained by the aerobic count (Table 2), this is not the case for the trials conducted by PEF. Here, directly after treatment, the reduction compared to the untreated samples was rather nominal. After 2 and 8 weeks, the microorganisms were not able to grow in higher numbers due to low pH. Despite the low microbial counts, contamination could cause a bombage/bulge of the product. The reasons for the growth of microorganisms in those samples could be (i) inhomogeneous treatment of the sample due to “cold spots” related to the electric field distribution within the treatment chamber (56) or (ii) recontamination during filling, since a continuous PEF-system with a co-linear treatment chamber was used for the trials.

**TABLE 3:** Counts of yeasts and molds of the stored sea buckthorn syrups depending on the pasteurization treatment.

Variety	Treatment	Yeasts and molds [CFU/mL]				
		0 Week	2 Weeks	4 Weeks	6 Weeks	8 Weeks
BO	untreated	152 ± 10	7*10^5^ ± 0	2*10^7^ ± 1.3*10^2^	1.3*10^7^ ± 1*10^2^	9*10^5^ ± 87*10^3^
LK	untreated	93 ± 15	<10^1^	<10^1^	3.55*10^5^ ± 2*10^3^	4.2*10^5^ ± 1*10^3^
BO	HPP 4 min	<10^1^	<10^1^	<10^1^	<10^1^	<10^1^
LK	HPP 4 min	<10^1^	<10^1^	<10^1^	<10^1^	<10^1^
BO	HPP 8 min	<10^1^	<10^1^	<10^1^	<10^1^	<10^1^
LK	HPP 8 min	<10^1^	<10^1^	<10^1^	<10^1^	<10^1^
BO	PEF	10 ± 1	122 ± 8	-	-	100 ± 5
LK	PEF	5 ± 2	90 ± 10	-	-	10 ± 1
BO	OH 85°C	<10^1^	<10^1^	<10^1^	<10^1^	<10^1^
LK	OH 85°C	<10^1^	<10^1^	<10^1^	<10^1^	<10^1^
BO	OH 91°C	<10^1^	<10^1^	<10^1^	<10^1^	<10^1^
LK	OH 91°C	<10^1^	<10^1^	<10^1^	<10^1^	<10^1^
BO	HF 88°C	<10^1^	<10^1^	<10^1^	<10^1^	<10^1^
LK	HF 88°C	<10^1^	<10^1^	<10^1^	<10^1^	<10^1^
BO	HF + C 88°C	<10^1^	<10^1^	<10^1^	<10^1^	<10^1^
LK	HF + C 88°C	<10^1^	<10^1^	<10^1^	<10^1^	<10^1^
BO	AF 80°C	<10^1^	<10^1^	<10^1^	<10^1^	<10^1^
LK	AF 80°C	<10^1^	<10^1^	<10^1^	<10^1^	<10^1^
BO	AF 87°C	<10^1^	<10^1^	<10^1^	<10^1^	<10^1^
LK	AF 87°C	<10^1^	<10^1^	<10^1^	<10^1^	<10^1^
BO	WB 89°C	<10^1^	<10^1^	<10^1^	<10^1^	<10^1^
LK	WB 89°C	<10^1^	<10^1^	<10^1^	<10^1^	<10^1^

WB (water bath, 89°C), AF (aseptic filling, 80–87.5°C), HF (hot filling at 88°C without cooling), HF + C (hot filling at 88°C plus cooling at 10°C), HPP (high pressure processing, 600 MPa/35°C/4 and 8 min), PEF (pulsed electric fields, 29.5 kV/cm, 6 μs, 100 Hz, Inlet 40°C and outlet 67°C), OH (ohmic heating, 0.8–0.9 kW, 85–91°C); LK (*Leikora*) and BO (*Botanica*).

In general, it can be concluded that, except for PEF, all other used technologies lead to a microbial free product over a storage period of 8 weeks if the samples are stored under chilled conditions (8°C). Therefore, the data are in general in accordance with previously reported data on the microbial storage stability in fruit juices. Timmermans et al. (45) stored orange juice (initial microbial content 10^3^–10^4^ CFU/ml) treated with HPP, PEF, and heat for 58 days at 4°C. They concluded that all samples treated with mild heat, HPP, and PEF showed to be below the detection limit after 2 months of storage at 4°C. The latter corresponds to expectations, since all process conditions were selected for equivalent processing, with respect to microbial food safety and spoilage. Yildiz et al. (57) conducted shelf-life tests with strawberry juice (initial microbial content 10^3^ CFU/ml) processed with HPP, ultrasound (US), PEF, and heat pasteurization. Here, a reduction of 2 log_10_ was achieved, except for PEF (growth after 28 days) and the untreated sample, no additional growth was monitored during the storage period of 42 days.

### 3.2. Color

All pasteurization treatments altered the color of the treated syrups, which increased slightly with the continued storage time. As indicated in Table 4, the smallest changes in comparison to the untreated sample (control) can be found for the HPP treated sample followed by PEF and thermal (WB, AF, HF, OH). When comparing the performance of the thermal process, the best color retention can be found for hot filling at 88°C as well as for the combination with cooling. HPP can retain the original color of the product while guaranteeing a microbial stable product. The trends found in this study are in consistent with the literature (57–60). There were no significant differences for the two syrups made from different varieties of sea buckthorn.

**TABLE 4:** Color changes indicated as ΔE-value of sea buckthorn syrup depending on treatment type and the storage time.

Variety:	LK			BO		
Treatment:	0 Month	1 Month	2 Months	0 Month	1 Month	2 Months
HPP 4 min	1.4^a^	1.9^a^	2.1^a^	1.1^a^	1.8^a^	2.0^a^
HPP 8 min	1.4^a^	1.8^a^	2.0^a^	1.2^a^	1.7^a^	2.1^a^
PEF	3.6^e^	3.8^b^	4.2^b^	3.7^e^	4.0^e,f^	4.3^c^
OH 85°C	3.33^c,d^	4.56^c^	6.19^f^	2.58^c,d^	3.37^c^	4.25^c^
OH 91°C	4.47^g^	4.81^d^	5.94^e^	2.25^b^	3.02^b^	5.53^f^
AF 80°C	3.45^d^	6.14^f^	7.1^g^	4.57^f^	4.34^g^	6.55^g^
AF 87°C	2.48^b^	5.43^e^	7.13^g^	2.76^d^	3.78^d^	5.26^f^
HF 88°C	3.18^c^	5.31^e^	5.23^c^	2.45^c^	3.90^d,e^	4.74^d^
HF + C 88°C	3.27^c^	4.81^d^	5.60^d^	2.22^b^	3.08^b^	3.74^b^
WB 89°C	3.9^f^	4.6^c,d^	5.2^c^	3.5^e^	4.2^f,g^	5.0^e^

Noticeable for the untrained human eye are ΔE differences > 2 (61–63); WB (water bath, 89°C), AF (aseptic filling, 80–87.5°C), HF (hot filling at 88°C without cooling), HF + C (hot filling at 88°C plus cooling at 10°C), HPP (high pressure processing, 600 MPa/35°C/4 and 8 min), PEF (pulsed electric fields, 29.5 kV/cm, 6 μs, 100 Hz, Inlet 40°C and outlet 67°C), OH (ohmic heating, 0.8–0.9 kW, 85–91°C); LK (*Leikora*) and BO (*Botanica*). Letters indicate grouping based on significant differences (*p* > 0.05) calculated with ANOVA. Values that do not share a letter are significant different. The ANOVA compared the technologies within one storage point.

The orange-yellow color of sea buckthorn syrup is mainly attributed to the high carotene content. Furthermore, sea buckthorn juice contains several types of particulates. Like tissue pieces and oil droplets (yellow) that contribute to the color. Clumps of different materials containing spherical droplets that were yellow-brown were frequently found (20, 64). Furthermore, a significant impact of different varieties of sea buckthorns on the color of the berries was previously reported by Tiitinen et al. (65).

In general, color changes in fruit juices after different preservation treatments were also reported by several other authors (12, 33, 45, 66–70). According to the results of the current study with sea buckthorn syrup, Wibowo et al. (33) reported only a slight color degradation of HPP treated cloudy apple juice directly after treatment. However, during storage, the formation of brown pigments was observed due to enzyme activity. Chen et al. (66) reported a weaker impact of HPP on the color pigments in pomegranate juice (chlorophyll, lycopenes, and anthocyanins) compared to a HTST treatment. Moreover, in orange juice, only a slight deviation (a growing trend in L* values) was observed compared to the untreated control juice after HPP treatment (71, 72). Timmermans et al. (45) compared the PEF, HPP, untreated and thermally pasteurized (tubular heat exchanger) orange juice directly after treatment, as well as after 60 days of storage at 4°C. The trends over the storage period are similar to those shown in this work. After processing, they did not observe such severe differences between the different technologies, especially the thermally treated samples, but this could be attributed to the nature of the juices and the lower temperatures used for the thermal treatment (72 vs. 80–88°C) by Timmermans et al. (45). However, the impact of HPP, PEF, and OH treatment on the color of juices was reported to differ significantly with respect to the types of juices. For some juice types, a higher color deviation by HPP and PEF was reported compared to conventional thermal treatments. These different observations could be due to the different interactions of the pigments with the HPP treatment, as well as enzymatic activity within the HPP and PEF treated juices (12, 45, 67, 68).

Similarly to the current study, no significant differences in color in orange and pineapple juice during conventional heating and OH were previously reported (69). However, other authors reported better color retention by OH compared to conventional thermal pasteurization treatments. Yildiz et al. (70) reported a reduced browning in pomegranate juice by OH compared to conventional heating. Based on these results, Lee et al. (73) reported a slightly better color retention in orange juices by OH compared to conventional heating.

The change in color during storage is dependent on the syrup and the selected treatment. Directly after the treatment, the innovative and gentler technologies such as OH, PEF, and HPP are superior to the heat treatment. Over longer storage periods (40–60 days), color changes can occur within the non-thermally treated juices, but this is highly dependent on the type of juice and the remaining enzymes that were not deactivated. For sea buckthorn, it was found that the HPP, PEF, and OH treatment are promising alternatives keeping changes to a minimum even for long storage periods compared to the thermal treatment.

### 3.3. Impact of treatments on bioactive compounds

Analysis of ascorbic acid, tocopherols, carotenoids, total phenolic compounds, and antioxidant activity was performed to assess the impact of pasteurization treatments on the quality of sea buckthorn syrup. The quality was monitored during an 8-week storage time, with analysis performed every 2 weeks. In total, 5 storage points were monitored including fresh syrup (untreated) and pasteurized syrup.

**FIGURE 1:**
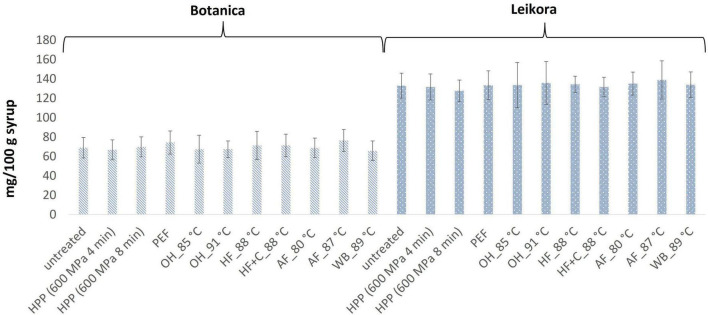
Ascorbic acid content in sea buckthorn syrup (mg AA/100 g fw). Values are mean, error bars represent 95% confidence interval. WB (water bath, 89°C), AF (aseptic filling, 80–87.5°C), HF (hot filling at 88°C without cooling), HF + C (hot filling at 88°C plus cooling at 10°C), HPP (high pressure processing, 600 MPa/35°C/4 and 8 min), PEF (pulsed electric fields, 29.5 kV/cm, 6 μs, 100 Hz, Inlet 40°C and outlet 67°C), OH (ohmic heating, 0.8–0.9 kW, 85–91°C); LK (*Leikora*) and BO (*Botanica*).

Analysis have shown that neither pasteurization nor storage time had an impact on the content of monitored bioactive compounds. Their stability during the treatments was probably caused by factors such as (i) low pH of the juice (in favor of ascorbic acid), (ii) higher viscosity due to high sugar content that limits oxidation, (iii) synergistic effect of carotenoids and tocopherols (74), and (iv) mild processing temperature. It is also known that sea buckthorn does not contain ascorbate oxidase, an enzyme responsible for the oxidation of ascorbic acid (75). Therefore, ascorbic acid was stable during processing and storage time. The mean content of ascorbic acid in *Leikora* variety treated syrups (Figure 1) was 134 ± 3 mg/100 g of syrup (untreated syrup 133 ± 6 mg/100 g) and *Botanica* variety treated syrups was 70 ± 3 mg/100 g of syrup (untreated syrup 69 ± 5 mg/100 g). The values were comparable with those presented in literature (13, 76). Our results confirmed also other studies, where different pasteurization conditions were applied to sea buckthorn juices. Seglina and Karklina (77) pasteurized sea buckthorn juices at temperatures 65, 75, and 85°C for 30, 20, and 10 min. They found out that the content of vitamin C did not change depending on the temperature and duration of pasteurization. The same observation was found also by Mezey et al. (76) who have studied the impact of (i) the modified VAT pasteurization process, presenting a single pasteurization (treatment at 85°C for 5 min) and the double pasteurization approach (treatment at 85°C for 5 min, then cooling down to 5°C and immediate treatment for 5 min at 85°C), and (ii) sterilization at 120°C for 3 min. However, worth to notice that some studies have also reported a significant loss of vitamin C. Manea and Buruleanu (78) observed an almost 86% decrease in vitamin C content during a 3-month storage period at refrigeration conditions in thermal pasteurized sea buckthorn juices. Another decrease in vitamin C content (11 to 12%) was observed during a 7-day storage time (at 6°C) of sea buckthorn juice treated by high-temperature short-time pasteurization (HTST, 90°C, 45 s) (79).

**FIGURE 2:**
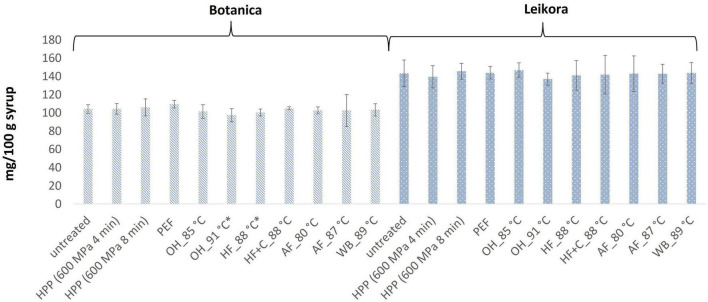
Antioxidant reducing capacity expressed as gallic acid equivalent in mg/100 g in sea buckthorn syrup. Values are mean, error bars represent 95% confidence interval. WB (water bath, 89°C), AF (aseptic filling, 80–87.5°C), HF (hot filling at 88°C without cooling), HF + C (hot filling at 88°C plus cooling at 10°C), HPP (high pressure processing, 600 MPa/35°C/4 and 8 min), PEF (pulsed electric fields, 29.5 kV/cm, 6 μs, 100 Hz, Inlet 40°C and outlet 67°C), OH (ohmic heating, 0.8–0.9 kW, 85–91°C); LK (*Leikora*) and BO (*Botanica*). Samples marked with *indicate significant differences between samples (*p* < 0.05).

**FIGURE 3:**
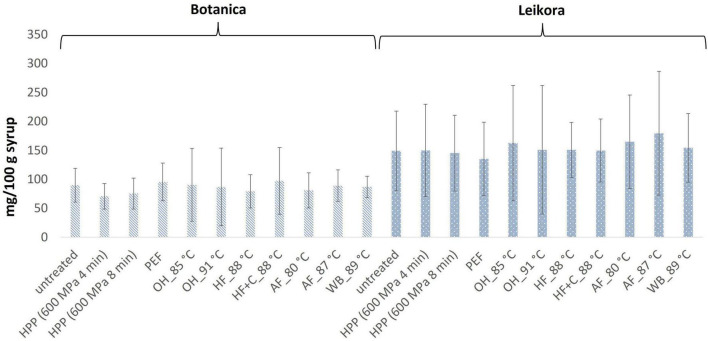
DPPH radical scavenging activity expressed as ascorbic acid equivalent in mg/100 g in sea buckthorn syrup. Values are mean, error bars represent 95% confidence interval. WB (water bath, 89°C), AF (aseptic filling, 80–87.5°C), HF (hot filling at 88°C without cooling), HF + C (hot filling at 88°C plus cooling at 10°C), HPP (high pressure processing, 600 MPa/35°C/4 and 8 min), PEF (pulsed electric fields, 29.5 kV/cm, 6 μs, 100 Hz, Inlet 40°C and outlet 67°C), OH (ohmic heating, 0.8–0.9 kW, 85–91°C); LK (*Leikora*) and BO (*Botanica*).

**FIGURE 4:**
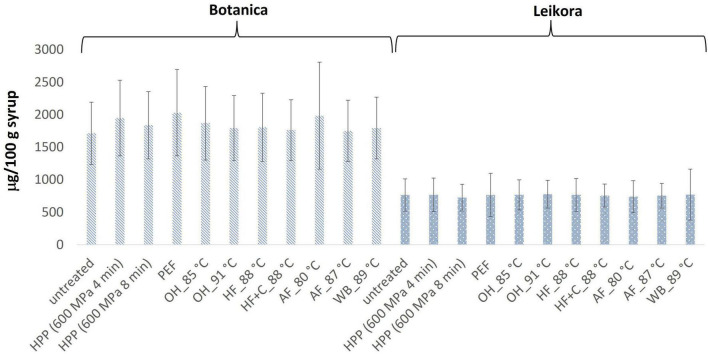
Vitamin E content in μg/100 g in sea buckthorn syrup. Values are mean, error bars represent 95% confidence interval. WB (water bath, 89°C), AF (aseptic filling, 80–87.5°C), HF (hot filling at 88°C without cooling), HF + C (hot filling at 88°C plus cooling at 10°C), HPP (high pressure processing, 600 MPa/35°C/4 and 8 min), PEF (pulsed electric fields, 29.5 kV/cm, 6 μs, 100 Hz, Inlet 40°C and outlet 67°C), OH (ohmic heating, 0.8–0.9 kW, 85–91°C); LK (*Leikora*) and BO (*Botanica*).

**FIGURE 5:**
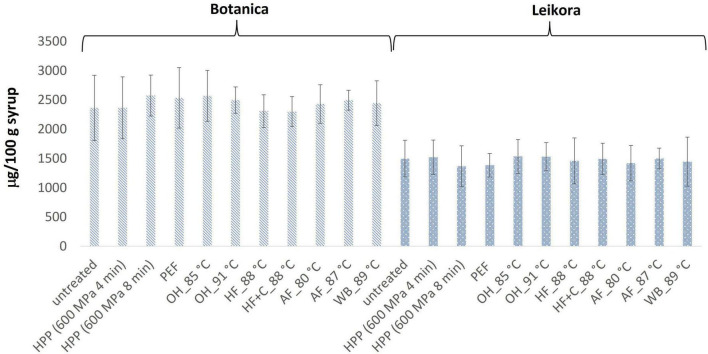
Carotenoids content in μg/100 g in sea buckthorn syrup. Values are mean, error bars represent 95% confidence interval. WB (water bath, 89°C), AF (aseptic filling, 80–87.5°C), HF (hot filling at 88°C without cooling), HF + C (hot filling at 88°C plus cooling at 10°C), HPP (high pressure processing, 600 MPa/35°C/4 and 8 min), PEF (pulsed electric fields, 29.5 kV/cm, 6 μs, 100 Hz, Inlet 40°C and outlet 67°C), OH (ohmic heating, 0.8–0.9 kW, 85–91°C); LK (*Leikora*) and BO (*Botanica*).

In general, the levels of bioactive compounds in both, *Botanica* and *Leikora*, varieties (Figures 1–5) differed according to the variety. The antioxidant activity determined employing the Folin-Ciocalteu assay and DPPH radical scavenging activity provided in the frame of each variety comparable results (*p* > 0.05). However, in two samples of variety *Botanica* (OH_91°C and HF_88°C), the antioxidant activity was slightly lower in comparison to PEF. The mean values observed in *Leikora* variety treated syrups were 154 ± 24 mg/100 g of ascorbic acid equivalent (untreated syrup 149 ± 68 mg/100 g of ascorbic acid equivalent) and 143 ± 6 mg/100 g of gallic acid equivalent (untreated syrup 143 ± 14 mg/100 g of gallic acid equivalent). Regarding the *Botanica* variety the mean antioxidant activity in treated syrups was determined as 85 ± 18 mg/100 g of ascorbic acid equivalent (untreated syrup 90 ± 30 mg/100 g of ascorbic acid equivalent) and 102 ± 10 mg/100 g of gallic acid equivalent (untreated syrup 104 ± 4 mg/100 g of gallic acid equivalent). Alexandrakis et al. (18) observed a slight increase in antioxidant activity (determined as DPPH radical scavenging capacity) when sea buckthorn juices were processed applying high pressure processing (HPP) at 200–600 MPa (ambient temperature 25°C). The authors explained this increase as the impact of high pressure disrupting the cell walls and releasing the intracellular antioxidant compounds. Higher processing temperatures (up to 35°C) did not significantly affect the antioxidant activity. A slight reduction (5%) in antioxidant activity was observed at sample treatment 600 MPa (35°C) for 5 min. However, the authors suggested these conditions to be optimal due to higher antioxidant retention and sufficient inactivation of pectin methyl esterase that have been investigated (18). The authors have also presented that conventional thermal pasteurization, already at 60°C for 1 min, decreased the antioxidant activity 2.5-fold compared to the untreated sample. While the ascorbic acid was almost twice higher in variety *Leikora*, the content of carotenoids (expressed as sum of β-carotene, lutein and zeaxanthin) and tocopherols (expressed as vitamin E activity) were found to be approximately twice lower (carotenoids, mean value of untreated syrup 1496 ± 312 μg/100 g syrup and treated syrups 1465 ± 120 μg/100 g syrup and; vitamin E, mean value of untreated syrup 763 ± 248 μg/100 g syrup and treated syrup 758 ± 32 μg/100 g syrup) compared to *Botanica* variety (carotenoids, mean value of untreated syrup 2362 ± 558 μg/100 g syrup and treated syrups 2451 ± 200 μg/100 g syrup and; vitamin E, mean value of untreated syrup 1714 ± 480 μg/100 g syrup and treated syrup 1857 ± 196 μg/100 g syrup). The content of these compounds was not affected by pasteurization (*p* > 0.05). Our results are confirmed by Seglina and Karklina (77) and Skąpska et al. (80) who investigated the impact of pasteurization on sea buckthorn juice. Similarly, as in our study, the carotenoid content was not affected. However, still very scare information describing the impact of mild pasteurization technologies on the content of carotenoids and vitamin E during the treatment of sea buckthorn juices or syrups is available. Therefore, future investigations should take place.

### 3.4. Impact of preservation treatment on the metabolomic fingerprint

**FIGURE 6:**
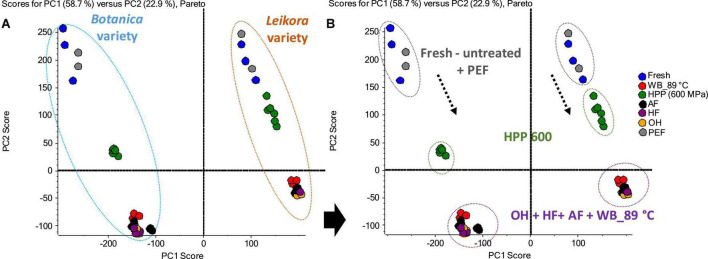
Principal component analysis (PCA) of sea buckthorn syrups. **(A)** Clustering according to the varieties, **(B)** clustering according to the treatments. WB (water bath, 89°C), AF (aseptic filling, 80–87.5°C), HF (hot filling at 88°C without cooling), HF + C (hot filling at 88°C plus cooling at 10°C), HPP (high pressure processing, 600 MPa/35°C/4 and 8 min), PEF (pulsed electric fields, 29.5 kV/cm, 6 μs, 100 Hz, Inlet 40°C and outlet 67°C), OH (ohmic heating, 0.8–0.9 kW, 85–91°C); LK (*Leikora*) and BO (*Botanica*).

The best to our knowledge, up to date, no metabolome study of sea buckthorn syrups treated under different preservation technologies has been published. Therefore, the impact of these preservation technologies was investigated in a comprehensive way, employing UHPLC-HRMS/MS. The generated data were evaluated and visualized employing Principal Component Analysis (PCA), where the principal component PC1 and PC2 together described 81.6% of the sample set variability (58.7 and 22.2% for PC1 and PC2, respectively). Clustering was specific for each variety (*Botanica* and *Leikora*) (Figure 6A). Furthermore, in the data set of each variety, the syrups clustered according to the type of pasteurization (Figure 6B). Fresh (untreated) syrups clustered with PEF treated syrups due to markers (compounds) that were similarly affected during the treatment. This shows that PEF due to the lower thermal load applied presents a mild technology that affected the metabolome insignificantly compared to ohmic heating (OH), high temperature short time treatment by aseptic filling (AF), hot filling (HF) or pasteurization in the water bath at 89°C (WB_89°C) that clustered together due to similar changes in their metabolome. However, high pressure processing (HPP) clustered separately, close to PEF, and untreated–fresh syrup.

**FIGURE 7:**
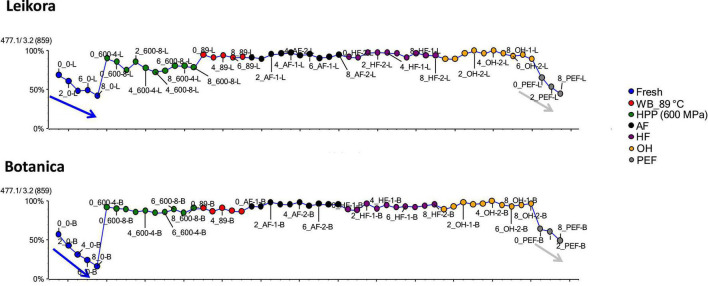
Variable plot presenting decrease of isorhamnetin glucoside in fresh (blue arrow) and PEF treated syrup (gray arrow) during storage time weeks 0, 2, 4, 6, and 8. WB (water bath, 89°C), AF (aseptic filling, 80–87.5°C), HF (hot filling at 88°C without cooling), HF + C (hot filling at 88°C plus cooling at 10°C), HPP (high pressure processing, 600 MPa/35°C/4 and 8 min), PEF (pulsed electric fields, 29.5 kV/cm, 6 μs, 100 Hz, Inlet 40°C and outlet 67°C), OH (ohmic heating, 0.8–0.9 kW, 85–91°C); LK (*Leikora*) and BO (*Botanica*).

**FIGURE 8:**
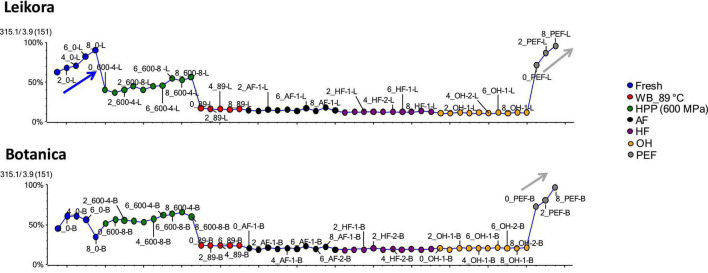
Variable plot showing the increase of aglycon isorhamnetin in fresh (blue arrow) and PEF treated syrup (gray arrow) during storage time weeks 0, 2, 4, 6, and 8. WB (water bath, 89°C), WB (water bath, 89°C), AF (aseptic filling, 80–87.5°C), HF (hot filling at 88°C without cooling), HF + C (hot filling at 88°C plus cooling at 10°C), HPP (high pressure processing, 600 MPa/35°C/4 and 8 min), PEF (pulsed electric fields, 29.5 kV/cm, 6 μs, 100 Hz, Inlet 40°C and outlet 67°C), OH (ohmic heating, 0.8–0.9 kW, 85–91°C); LK (*Leikora*) and BO (*Botanica*).

**FIGURE 9:**
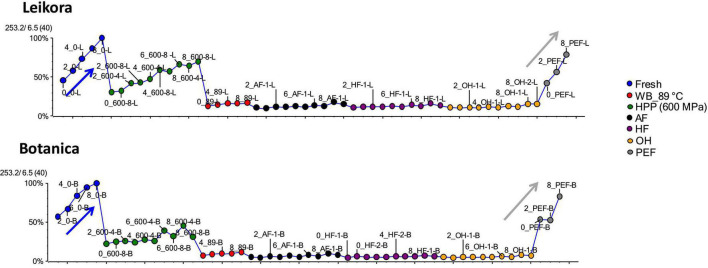
Variable plot showing the increase of palmitoleic acid in fresh (blue arrow) and PEF treated syrup (gray arrow) during storage time weeks 0, 2, 4, 6, and 8. WB (water bath, 89°C), AF (aseptic filling, 80–87.5°C), HF (hot filling at 88°C without cooling), HF + C (hot filling at 88°C plus cooling at 10°C), HPP (high pressure processing, 600 MPa/35°C/4 and 8 min), PEF (pulsed electric fields, 29.5 kV/cm, 6 μs, 100 Hz, Inlet 40°C and outlet 67°C), OH (ohmic heating, 0.8–0.9 kW, 85–91°C); LK (*Leikora*) and BO (*Botanica*).

**FIGURE 10:**
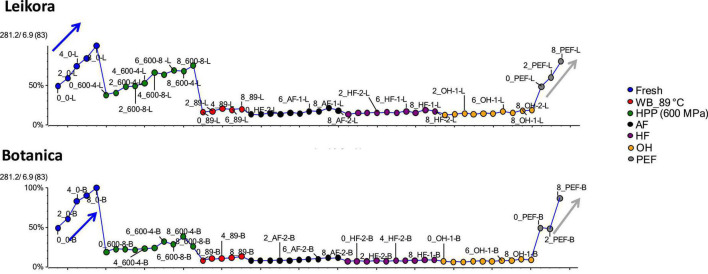
Variable plot showing the increase of oleic acid in fresh (blue arrow) and pulsed electric fields (PEF) treated syrup (gray arrow) during storage time weeks 0, 2, 4, 6, and 8. WB (water bath, 89°C), AF (aseptic filling, 80–87.5°C), HF (hot filling at 88°C without cooling), HF + C (hot filling at 88°C plus cooling at 10°C), HPP (high pressure processing, 600 MPa/35°C/4 and 8 min), PEF (pulsed electric fields, 29.5 kV/cm, 6 μs, 100 Hz, Inlet 40°C and outlet 67°C), OH (ohmic heating, 0.8–0.9 kW, 85–91°C); LK (*Leikora*) and BO (*Botanica*).

Specific markers that were identified to contribute to the clustering of groups, were bounded flavonoid glycosides that were found in higher amounts, especially in thermally treated syrups, in contrast with their free aglycons, which were more abundant in non-thermally treated syrups (fresh, PEF, and HPP). The identified flavonoids (presented in Supplementary Table 1) were in majority glycosylated forms of aglycones of isorhamnetin, quercetin and kaempferol. This is in accordance with a review presented by Ciesarová et al. (13). While a decrease in isorhamnetin glucoside in fresh and PEF treated syrup over an 8-week storage time was observed (Figure 7) an increase in its aglycone isorhamnetin during the same storage time was observed (Figure 8). Interestingly, a similar trend was also observed in the case of free fatty acids (e.g., palmitoleic acid or oleic acid, Figures 9, 10) that were dominant in non-thermally treated syrups (fresh, PEF, and HPP). These free fatty acids were presented to be the most abundant in sea buckthorn. In some varieties depending on the origin of the plant, palmitoleic may present even 20–45% of the total fatty acids (81). Other identified free fatty acids were palmitic acid, oleic acid, linoleic and linolenic acid (Supplementary Table 1). The free fatty acids and aglycons increased according to storage time. This indicated that the PEF and HPP treatment, enzymes remained activated and were not denaturized as during thermal treatments. These enzymes, most probably glucosidases and lipases, were responsible for the catalyzation of hydrolytic cleavage of glyosidic and ester bounds. To inactivate the enzyme activity, in case of PEF, higher electric field (above 40 kV/cm), short heating after treatment or fast cooling have shown to be effective. For the HPP treatment, probably longer processing time in combination with slightly higher temperatures will contribute to the enzyme inactivation. Although, this is highly dependent on the investigated enzyme (82, 83).

### 3.5. Sensory evaluation

**FIGURE 11:**
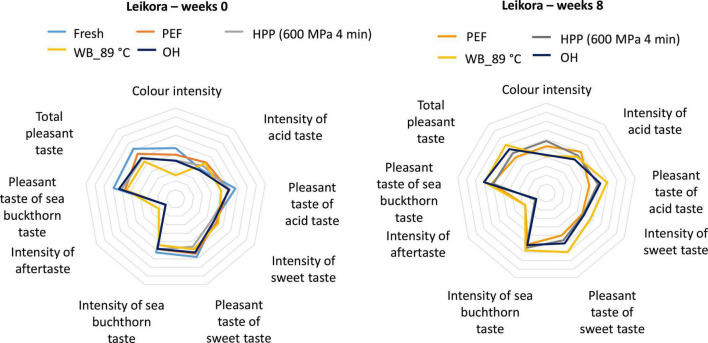
Results of the sensory evaluation, syrup prepared from *Leikora* variety. Week 0–represents the syrup after treatment, Weeks 8–the end of the storage period [Fresh–untreated (light blue), PEF–Pulsed electric field (orange), HPP–high pressure processing at 600 MPa, 4 min (gray), WB_89°C–pasteurization in water bath at 89°C (yellow), OH–Ohmic heating (dark blue)].

**FIGURE 12:**
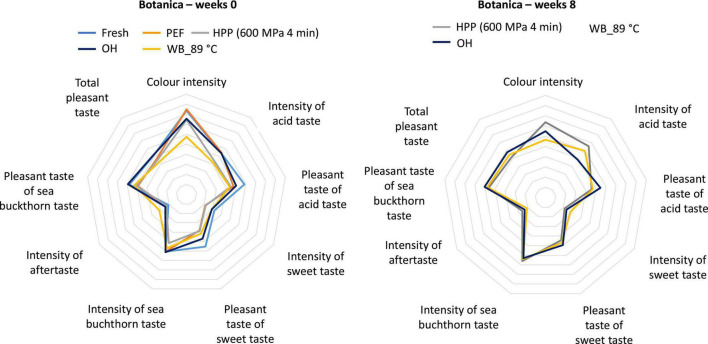
Results of sensory evaluation, syrup prepared from *Botanica* variety. Week 0–represents syrup after treatment, Weeks 8–the end of storage period [Fresh–untreated (light blue), PEF–Pulsed electric field (orange), HPP–high pressure processing at 600 MPa, 4 min (gray), WB_89°C–pasteurization in water bath at 89°C (yellow), OH–Ohmic heating (dark blue)].

Prior to sensory analysis, the syrups were subjected to microbiological evaluation to ensure product safety (see Section “3.1. Microbiological inactivation”). Syrups were sensorially evaluated at week 0 and the results were compared with syrups evaluated after an 8-week storage period (Figures 11, 12). As fresh or untreated samples (both varieties) and PEF treated samples (*Botanica*) were not shelf stable during the whole storage period, they were not considered for the sensory evaluation after 8 weeks. No significant differences were found after 8 weeks of storage. However, it should be noted that the color was favorably rated (comparable to fresh syrup) for PEF and HPP treated syrups prepared from both varieties *Botanica* and *Leikora*. The color intensity, after an 8-week storage time, was not significantly different for both varieties treated with OH. Syrup (*Leikora* variety) pasteurized in the water bath at 89°C was positively evaluated after 8 weeks regarding color intensity, sea buckthorn taste, pleasant acid taste, and sweet taste. Regarding the syrups prepared from the *Botanica* variety, the intensity of the acid taste was more pronounced after 8 weeks of storage time in the syrup treated in the water bath at 89°C and by HPP. In general, an increase in intensity of the sea buckthorn taste and a total pleasant taste was observed in all the tested syrups. In addition, a sequence and preference tests were performed with syrups stored for 8 weeks. In both tests, the HPP treated syrup was more in favor. In the sequence test, the HPP treated syrup ranked 1st, followed by ohmic heating, conventional pasteurization at 89°C and PEF treatment. Regarding the preference test 67% evaluators favorably evaluated the HPP treated syrup. The impact of conventional and novel preservation technologies on the sensory attributes of fruit juices like apple juice (44, 84), orange juice (38) or water melon juice (85) was already investigated in the literature. Several authors reported that PEF and HPP treated juices were received more similar to conventional heated juices (38, 84). These findings are however only partly in accordance with the results obtained in this study.

Several studies are available presenting sensory evaluation of sea buckthorn juices reviewed in Ciesarová et al. (13). However, up to date there are no study assessing the sensory quality of sea buckthorn syrups treated under different pasteurization conditions as well with respect to storage time.

## 4. Conclusion and outlook

In this study, for the first time, both targeted and non-targeted (fingerprinting) analyses were employed to investigate the impact of 3 different conventional thermal (AF, HF, WB) one innovative thermal (OH) and two non-thermal (PEF and HPP) preservation technologies on the nutritional parameters and the sensorial quality profile of a sea buckthorn syrup. All applied treatments, except for the PEF treatment, lead to a microbial safe and stable syrup. The targeted analyses of bioactive compounds (ascorbic acid, total phenolics, total antioxidants expressed as antioxidative capacity, tocopherols, and carotenoids) did not document a significant impact of the various treatments or the storage duration. This was probably due to (i) low pH of the syrup (in favor of ascorbic acid), (ii) higher viscosity due to the high sugar content limiting oxidation, (iii) synergic effect of carotenoids and tocopherols, and (iv) mild processing temperature. It is worth noting that significant differences in bioactive compounds content between the two sea buckthorn varieties *Leikora* and *Botanica* were observed. Similarly, U-HPLC-HRMS/MS based metabolomic fingerprints fairly differed. The analysis of generated data by advanced statistical methods showed clustering according to the way of preservation for both types of syrups and respective marker compounds were identified. Interestingly, compared to other tested technologies, higher content of free fatty acids and increased amount of flavonoid aglycones, were observed in case of stored PEF treated syrups, what suggests preserving activity of native enzymes such as lipases and glycosidases. In any case, further validation of the current results would be required for the purpose of practical application. The usage of targeted and untargeted chemical analyses leads to a metabolomic profile of the food and shows the history of the food. In the future these kind of approaches in combination with more knowledge of the applied processing technologies could be used to evaluate food authenticity, and treatment history of the food. Our results indicate that the flavonoids in sea buckthorn syrup could be used as an indicator compound to determine the technology used and therefore give indications about the food quality, the process applied, and also prevent potential food fraud. An open research question, which remains is, if these findings can be applied to other juices or, if there are other target compounds involved. Further, research on this topic including juices and syrups from other fruits could give valuable further insights.

## Data availability statement

The original contributions presented in this study are included in this article/Supplementary material, further inquiries can be directed to the corresponding author.

## Author contributions

RS, MG, and BH were the main authors of this manuscript. FS, TT, and TF helped with trials and the design of the experiments from BOKU. HJ head of the department at BOKU supported the trials and revised the manuscript. CR head of the department at TU Berlin supported the trials and revised the manuscript. JH head of the department at UCT supported the trials and revised the manuscript. MT, LC, and KH helped with analysis and the design of the experiments from UCT. All authors contributed to the article and approved the submitted version.

## Footnotes

1
https://metlin.scripps.edu/

## References

[1] Bigliardi B Galati F. Innovation trends in the food industry: the case of functional foods. *Trends Food Sci Technol.* (2013) 31:118–29. 10.1016/j.tifs.2013.03.006

[2] Siró I Kápolna E Kápolna B Lugasi A. Functional food. Product development, marketing and consumer acceptance—a review. *Appetite.* (2008) 51:456–67. 10.1016/j.appet.2008.05.060 18582508

[3] Lucas BF Costa JAV Brunner TA. Superfoods: drivers for consumption. *J Food Prod Mark.* (2021) 27:1–9. 10.1080/10454446.2020.1869133

[4] Menrad K. Market and marketing of functional food in Europe. *J Food Eng.* (2003) 56:181–8. 10.1016/S0260-8774(02)00247-9

[5] Proestos C. Superfoods: recent data on their role in the prevention of diseases. *Curr Res Nutr Food Sci J.* (2018) 6:576–93. 10.12944/CRNFSJ.6.3.02

[6] Ghoshal G. Chapter 2 - emerging food processing technologies. In: Grumezescu AM Holban AM editors. *Food processing for increased quality and consumption, handbook of food bioengineering.* Cambridge: Academic Press (2018). p. 29–65. 10.1016/B978-0-12-811447-6.00002-3

[7] Horvat A Granato G Fogliano V Luning PA. Understanding consumer data use in new product development and the product life cycle in European food firms – an empirical study. *Food Qual Prefer.* (2019) 76:20–32. 10.1016/j.foodqual.2019.03.008

[8] Knorr D Watzke H. Food processing at a crossroad. *Front Nutr.* (2019) 6:85. 10.3389/fnut.2019.00085 31294027 PMC6603079

[9] Pinela J Ferreira ICFR. Nonthermal physical technologies to decontaminate and extend the shelf-life of fruits and vegetables: trends aiming at quality and safety. *Crit Rev Food Sci Nutr.* (2017) 57:2095–111. 10.1080/10408398.2015.1046547 26192014

[10] Caswell H. The role of fruit juice in the diet: an overview. *Nutr Bull.* (2009) 34:273–88. 10.1111/j.1467-3010.2009.01760.x

[11] Rajauria G Tiwari BK. Fruit juices. In: Rajauria G Tiwari BK editors. *Fruit juices.* Amsterdam: Elsevier (2018). p. 3–13. 10.1016/B978-0-12-802230-6.00001-1

[12] de Souza VR Popović V Bissonnette S Ros I Mats L Duizer L Quality changes in cold pressed juices after processing by high hydrostatic pressure, ultraviolet-c light and thermal treatment at commercial regimes. *Innov Food Sci Emerg Technol.* (2020) 64:102398. 10.1016/j.ifset.2020.102398

[13] Ciesarová Z Murkovic M Cejpek K Kreps F Tobolková B Koplík R Why is sea buckthorn (*Hippophae rhamnoides* L.) so exceptional? a review. *Food Res Int.* (2020) 133:109170. 10.1016/j.foodres.2020.109170 32466930

[14] Dong K Binosha Fernando WMAD Durham R Stockmann R Jayasena V. Nutritional value, health-promoting benefits and food application of sea *Buckthorn*. *Food Rev Int.* (2021) 2008:1–16. 10.1080/87559129.2021.1943429

[15] Koskovac M Cupara S Kipic M Barjaktarevic A Milovanovic O Kojicic K Sea buckthorn oil—a valuable source for cosmeceuticals. *Cosmetics.* (2017) 4:40. 10.3390/cosmetics4040040

[16] Suryakumar G Gupta A. Medicinal and therapeutic potential of Sea *Buckthorn* (*Hippophae rhamnoides* L.). *J Ethnopharmacol.* (2011) 138:268–78. 10.1016/j.jep.2011.09.024 21963559

[17] RHS Gardening. *Hippophae rhamnoides ‘Leikora’ (f/F).* (2021). Available online at: https://www.rhs.org.uk/plants/90871/hippophae-rhamnoides-leikora-(f-f)/details (accessed November 22, 2021).

[18] Alexandrakis Z Kyriakopoulou K Katsaros G Krokida M Taoukis P. Selection of process conditions for high pressure pasteurization of sea buckthorn juice retaining high antioxidant activity. *Food Bioproc Tech.* (2014) 7:3226–34. 10.1007/s11947-014-1299-5

[19] Bal LM Meda V Naik SN Satya S. Sea buckthorn berries: a potential source of valuable nutrients for nutraceuticals and cosmoceuticals. *Food Res Int.* (2011) 44:1718–27. 10.1016/j.foodres.2011.03.002

[20] Beveridge T Li TSC Oomah BD Smith A. Sea *Buckthorn* products: manufacture and composition. *J Agric Food Chem.* (1999) 47:3480–8. 10.1021/jf981331m 10552673

[21] Kaur C Kapoor HC. Antioxidants in fruits and vegetables – the millennium’s health. *Int J Food Sci Technol.* (2001) 36:703–25. 10.1111/j.1365-2621.2001.00513.x

[22] Vilas-Franquesa A Saldo J Juan B. Potential of sea buckthorn-based ingredients for the food and feed industry – a review. *Food Prod Process Nutr*. (2020) 2:17. 10.1186/s43014-020-00032-y

[23] Jiménez-Sánchez C Lozano-Sánchez J Segura-Carretero A Fernández-Gutiérrez A. Alternatives to conventional thermal treatments in fruit-juice processing. Part 1: techniques and applications. *Crit Rev Food Sci Nutr.* (2017) 57:501–23. 10.1080/10408398.2013.867828 25849158

[24] Deliza R Rosenthal A Abadio FBD Silva CHO Castillo C. Application of high pressure technology in the fruit juice processing: benefits perceived by consumers. *J Food Eng.* (2005) 67:241–6. 10.1016/j.jfoodeng.2004.05.068

[25] Knorr D Froehling A Jaeger H Reineke K Schlueter O Schoessler K. Emerging technologies in food processing. *Annu Rev Food Sci Technol.* (2011) 2:203–35. 10.1146/annurev.food.102308.124129 22129381

[26] Augusto PED Tribst AAL Cristianini M. High hydrostatic pressure and high-pressure homogenization processing of fruit juices. In: Rajauria G Tiwari BK editors. *Fruit Juices.* Amsterdam: Elsevier (2018). p. 393–421. 10.1016/B978-0-12-802230-6.00020-5

[27] Hendrickx MEG Knorr D editors. *Ultra high pressure treatment of foods, food engineering series.* Berlin: Springer (2001). 10.1007/978-1-4615-0723-9

[28] Barba FJ Parniakov O Pereira SA Wiktor A Grimi N Boussetta N Current applications and new opportunities for the use of pulsed electric fields in food science and industry. *Food Res Int.* (2015) 77:773–98. 10.1016/j.foodres.2015.09.015

[29] Icier F. Ohmic heating of fluid foods. In: Cullen P Tiwari B Valdramidis V editors. *Novel thermal and non-thermal technologies for fluid foods.* San Diego: Academic Press (2012). p. 305–67. 10.1016/B978-0-12-381470-8.00011-6

[30] Jaeger H Roth A Toepfl S Holzhauser T Engel KH Knorr D Opinion on the use of ohmic heating for the treatment of foods. *Trends Food Sci Technol.* (2016) 55:84–97. 10.1016/j.tifs.2016.07.007

[31] Leizerson S Shimoni E. Stability and sensory shelf life of orange juice pasteurized by continuous ohmic heating. *J Agric Food Chem.* (2005) 53:4012–8. 10.1021/jf047857q 15884832

[32] Timmermans RAH Mastwijk HC Berendsen LBJM Nederhoff AL Matser AM Van Boekel MAJS Moderate intensity pulsed electric fields (PEF) as alternative mild preservation technology for fruit juice. *Int J Food Microbiol.* (2019) 298:63–73. 10.1016/j.ijfoodmicro.2019.02.015 30925357

[33] Wibowo S Essel EA De Man S Bernaert N Van Droogenbroeck B Grauwet T Comparing the impact of high pressure, pulsed electric field and thermal pasteurization on quality attributes of cloudy apple juice using targeted and untargeted analyses. *Innov Food Sci Emerg Technol.* (2019) 54:64–77. 10.1016/j.ifset.2019.03.004

[34] Yildiz S Pokhrel P Unluturk S Barbosa-Cánovas G. Identification of equivalent processing conditions for pasteurization of strawberry juice by high pressure, ultrasound, and pulsed electric fields processing. *Innov Food Sci Emerg Technol.* (2019) 57:102195.

[35] Esteki M Shahsavari Z Simal-Gandara J. Gas chromatographic fingerprinting coupled to chemometrics for food authentication. *Food Rev Int.* (2020) 36:384–427. 10.1080/87559129.2019.1649691

[36] Kessler HG. *Food and bio process engineering: dairy technology.* Munich: Kessler (2002).

[37] Aganovic K Hertel C Vogel RF Johne R Schlüter O Schwarzenbolz U Aspects of high hydrostatic pressure food processing: perspectives on technology and food safety. *Compr Rev Food Sci Food Saf.* (2021) 20:3225–66. 10.1111/1541-4337.12763 34056857

[38] Buckow R Ng S Toepfl S. Pulsed electric field processing of orange juice: a review on microbial, enzymatic, nutritional, and sensory quality and stability: PEF preservation of orange juice. *Compr Rev Food Sci Food Saf.* (2013) 12:455–67. 10.1111/1541-4337.12026 33412665

[39] Saldaña G Álvarez I Condón S Raso J. Microbiological aspects related to the feasibility of PEF technology for food pasteurization. *Crit Rev Food Sci Nutr.* (2014) 54:1415–26. 10.1080/10408398.2011.638995 24580538

[40] Balasubramaniam V. Process development of high pressure-based technologies for food: research advances and future perspectives. *Curr Opin Food Sci.* (2021) 42:270–7. 10.1016/j.cofs.2021.10.001

[41] Sevenich R Rauh C Knorr D. Overview of research needs, future and potential applications of high-pressure processing. In: Knoerzer K Muthukumarappan K editors. *Innovative food processing technologies.* Amsterdam: Elsevier (2021). p. 1–18. 10.1016/B978-0-08-100596-5.22991-0

[42] Reineke K Schottroff F Meneses N Knorr D. Sterilization of liquid foods by pulsed electric fields – an innovative ultra-high temperature process. *Front Microbiol.* (2015) 6:400. 10.3389/fmicb.2015.00400 25999930 PMC4422003

[43] Meneses N Jaeger H Knorr D. Minimization of thermal impact by application of electrode cooling in a co-linear PEF treatment chamber. *J Food Sci.* (2011) 76:E536–43. 10.1111/j.1750-3841.2011.02368.x 22417588

[44] Aguilar-Rosas SF Ballinas-Casarrubias ML Nevarez-Moorillon GV Martin-Belloso O Ortega-Rivas E. Thermal and pulsed electric fields pasteurization of apple juice: effects on physicochemical properties and flavour compounds. *J Food Eng.* (2007) 83:41–6. 10.1016/j.jfoodeng.2006.12.011

[45] Timmermans RAH Mastwijk HC Knol JJ Quataert MCJ Vervoort L der Plancken IV Comparing equivalent thermal, high pressure and pulsed electric field processes for mild pasteurization of orange juice. Part I: impact on overall quality attributes. *Innov Food Sci Emerg Technol.* (2011) 12:235–43. 10.1016/j.ifset.2011.05.001

[46] Liu F Wang Y Li R Bi X Liao X. Effects of high hydrostatic pressure and high temperature short time on antioxidant activity, antioxidant compounds and color of mango nectars. *Innov Food Sci Emerg Technol.* (2014) 21:35–43. 10.1016/j.ifset.2013.09.015

[47] Sharma G Bala R. *Digital color imaging handbook, electrical engineering & applied signal processing series.* Boca Raton: CRC Press (2017).

[48] Bhave A Schulzova V Chmelarova H Mrnka L Hajslova J. Assessment of rosehips based on the content of their biologically active compounds. *J Food Drug Anal.* (2017) 25:681–90. 10.1016/j.jfda.2016.12.019 28911653 PMC9328834

[49] Hurkova K Uttl L Rubert J Navratilova K Kocourek V Stranska-Zachariasova M Cranberries versus lingonberries: a challenging authentication of similar *Vaccinium* fruit. *Food Chem.* (2019) 284:162–70. 10.1016/j.foodchem.2019.01.014 30744842

[50] Bevilacqua A Petruzzi L Perricone M Speranza B Campaniello D Sinigaglia M Nonthermal technologies for fruit and vegetable juices and beverages: overview and advances. *Compr Rev Food Sci Food Saf.* (2018) 17:2–62. 10.1111/1541-4337.12299 33350062

[51] Cullen PJ Brijesh kumar T Valdramidis V. *Novel thermal and non-thermal technologies for fluid foods.* Cambridge: Academic Press (2012). 10.1016/C2009-0-61145-4

[52] Jaeger H Knorr D Meneses N Reineke K Schlueter O. Food safety: shelf life extension technologies. In: Van Alfen NK editor. *Encyclopedia of agriculture and food systems.* Cambridge: Academic Press (2014). p. 289–303. 10.1016/B978-0-444-52512-3.00050-4

[53] Knorr D. Emerging technologies: back to the future. *Trends Food Sci Technol.* (2018) 76:119–23. 10.1016/j.tifs.2018.03.023

[54] Reineke K Mathys A. Endospore inactivation by emerging technologies: a review of target structures and inactivation mechanisms. *Annu Rev Food Sci Technol.* (2020) 11:255–74. 10.1146/annurev-food-032519-051632 31905011

[55] Yildiz S Pokhrel P Unluturk S Barbosa-Cánovas G. Changes in quality characteristics of strawberry juice after equivalent high pressure, ultrasound, and pulsed electric fields processes. *Food Eng Rev.* (2021) 13:601–12. 10.1007/s12393-020-09250-z 33648266

[56] Jaeger H Meneses N Knorr D. Impact of PEF treatment inhomogeneity such as electric field distribution, flow characteristics and temperature effects on the inactivation of E. coli and milk alkaline phosphatase. *Innov Food Sci Emerg Technol.* (2009) 10:470–80.

[57] Yildiz S Pokhrel PR Unluturk S Barbosa-Cánovas GV. Shelf life extension of strawberry juice by equivalent ultrasound, high pressure, and pulsed electric fields processes. *Food Res Int.* (2021) 140:110040. 10.1016/j.foodres.2020.110040 33648266

[58] Bull MK Zerdin K Howe E Goicoechea D Paramanandhan P Stockman R The effect of high pressure processing on the microbial, physical and chemical properties of *Valencia and Navel orange* juice. *Innov Food Sci Emerg Technol.* (2004) 5:135–49.

[59] Guo M Jin TZ Geveke DJ Fan X Sites JE Wang L. Evaluation of microbial stability, bioactive compounds, physicochemical properties, and consumer acceptance of pomegranate juice processed in a commercial scale pulsed electric field system. *Food Bioproc Tech.* (2014) 7:2112–20. 10.1007/s11947-013-1185-6

[60] Varela-Santos E Ochoa-Martinez A Tabilo-Munizaga G Reyes JE Pérez-Won M Briones-Labarca V Effect of high hydrostatic pressure (HHP) processing on physicochemical properties, bioactive compounds and shelf-life of pomegranate juice. *Innov Food Sci Emerg Technol.* (2012) 13:13–22. 10.1016/j.ifset.2011.10.009

[61] Witzel RF Burnham RW Onley JW. Threshold and suprathreshold perceptual color differences. *JOSA.* (1973) 63:615–25. 10.1364/JOSA.63.000615 4731340

[62] Wright WD. A re-determination of the trichromatic coefficients of the spectral colours. *Trans Opt Soc.* (1929) 30:141. 10.1088/1475-4878/30/4/301

[63] Wyszecki G Fielder GH. New color-matching ellipses. *JOSA.* (1971) 61:1135–52. 10.1364/JOSA.61.001135 5121883

[64] Beveridge T Harrison JE. Microscopic structural components of sea buckthorn (*Hippophae rhamnoides* L.) juice prepared by centrifugation. *LWT Food Sci Technol.* (2001) 34:458–61. 10.1006/fstl.2001.0791

[65] Tiitinen KM Hakala MA Kallio HP. Quality components of sea *Buckthorn* (*Hippophaë rhamnoides*) varieties. *J Agric Food Chem.* (2005) 53:1692–9. 10.1021/jf0484125 15740060

[66] Chen D Xi H Guo X Qin Z Pang X Hu X Comparative study of quality of cloudy pomegranate juice treated by high hydrostatic pressure and high temperature short time. *Innov Food Sci Emerg Technol.* (2013) 19:85–94. 10.1016/j.ifset.2013.03.003

[67] Jiménez-Sánchez C Lozano-Sánchez J Segura-Carretero A. Alternatives to conventional thermal treatments in fruit-juice processing. Part 2: effect on composition, phytochemical content, and physicochemical, rheological, and organoleptic properties of fruit juices. *Crit Rev Food Sci Nutr.* (2017) 57:637–52. 10.1080/10408398.2014.914019 25894933

[68] Oey I Lille M Van Loey A Hendrickx M. Effect of high-pressure processing on colour, texture and flavour of fruit- and vegetable-based food products: a review. *Trends Food Sci. Technol.* (2008) 19:320–8. 10.1016/j.tifs.2008.04.001

[69] Tumpanuvatr T Jittanit W. The temperature prediction of some botanical beverages, concentrated juices and purees of orange and pineapple during ohmic heating. *J Food Eng.* (2012) 113:226–33. 10.1016/j.jfoodeng.2012.05.044

[70] Yildiz H Bozkurt H Icier F. Ohmic and conventional heating of pomegranate juice: effects on rheology, color, and total phenolics. *Food Sci Technol Int.* (2009) 15:503–12.

[71] Hartyáni P Dalmadi I Knorr D. Electronic nose investigation of *Alicyclobacillus* acidoterrestris inoculated apple and orange juice treated by high hydrostatic pressure. *Food Control.* (2013) 32:262–9. 10.1016/j.foodcont.2012.10.035

[72] Hartyáni P Dalmadi I Cserhalmi Z Kántor DB Tóth-Markus M Sass-Kiss Á. Physical–chemical and sensory properties of pulsed electric field and high hydrostatic pressure treated citrus juices. *Innov Food Sci Emerg Technol.* (2011) 12:255–60.

[73] Lee SY Sagong HG Ryu S Kang DH. Effect of continuous ohmic heating to inactivate *Escherichia coli* O157:H7, *Salmonella Typhimurium* and *Listeria* monocytogenes in orange juice and tomato juice. *J Appl Microbiol.* (2012) 112:723–31. 22292508 10.1111/j.1365-2672.2012.05247.x

[74] Palozza P Krinsky NI. β-Carotene and α-tocopherol are synergistic antioxidants. *Arch Biochem Biophys.* (1992) 297:184–7. 10.1016/0003-9861(92)90658-J 1637180

[75] Seglina D Karklina D Ruisa S Krasnova I. The effect of processing on the composition of sea buckthorn juice. *J Fruit Ornam Plant Res.* (2006) 14:257.

[76] Mezey J Hegedûs O Mezeyová I Szarka K Hegedûsová A. Thermal treatment influence on selected nutritional values of common sea *Buckthorn* (*Hyppophae rhamnoides*) juice. *Agronomy.* (2022) 12:1834. 10.3390/agronomy12081834

[77] Seglina D Karklina D. The dynamics of vitamin C and total carotenes content in pasteurized sea-buckthorn juice. *Res Rural Dev.* (2005) 1:205–7.

[78] Manea I Buruleanu L. Mathematical model for the evaluation of the sea-buckthorn juice preservation. *Ovidius Univ Ann Chem.* (2009) 20:83–6.

[79] Gutzeit D Baleanu G Winterhalter P Jerz G. Vitamin C content in sea *Buckthorn* berries (*Hippophae rhamnoides* L. ssp rhamnoides) and related products: a kinetic study on storage stability and the determination of processing effects. *J Food Sci.* (2008) 73:C615–20. 10.1111/j.1750-3841.2008.00957.x 19021790

[80] Skąpska S Marszałek K Woźniak Ł Szczepańska J Danielczuk J Zawada K. The development and consumer acceptance of functional fruit-herbal beverages. *Foods Basel Switz.* (2020) 9:1819. 10.3390/foods9121819 33302360 PMC7762522

[81] Li TSC Beveridge THJ. *Sea Buckthorn (Hippophae rhamnoides L.): Production and Utilization*. Ottawa, ON: NRC Research Press (2003).

[82] Li YQ Chen Q Liu XH Chen ZX. Inactivation of soybean lipoxygenase in soymilk by pulsed electric fields. *Food Chem.* (2008) 109:408–14. 10.1016/j.foodchem.2008.01.001 26003365

[83] Toepfl S Siemer C Saldaña-Navarro G Heinz V. Chapter 6 - overview of pulsed electric fields processing for food. In: Sun DW editor. *Emerging technologies for food processing (second edition).* San Diego: Academic Press (2014). p. 93–114. 10.1016/B978-0-12-411479-1.00006-1

[84] Lee PY Kebede BT Lusk K Mirosa M Oey I. Investigating consumers’ perception of apple juice as affected by novel and conventional processing technologies. *Int J Food Sci Technol.* (2017) 52:2564–71. 10.1111/ijfs.13542

[85] Aganovic K Grauwet T Siemer C Toepfl S Heinz V Hendrickx M Headspace fingerprinting and sensory evaluation to discriminate between traditional and alternative pasteurization of watermelon juice. *Eur Food Res Technol.* (2016) 242:787–803. 10.1007/s00217-015-2586-8

