# Supplementary material for: Differentiation of sea buckthorn syrups processed by high pressure, pulsed electric fields, ohmic heating, and thermal pasteurization based on quality evaluation and chemical fingerprinting
Source: Front Nutr. 2023 Feb 14;10:912824. doi: 10.3389/fnut.2023.912824 (PMC9971502; doi:10.3389/fnut.2023.912824)
Supplement: Supplementary file 1 [file Table_1.docx]

Table 1: Major flavonoids and fatty acid identified by U-HPLC-HRMS/MS

| **Compound** | **Molecular formula** | **Ionization mode** | **Adduct** | ***m/z*** | **RT (min)** |
| --- | --- | --- | --- | --- | --- |
| Isorhamnetin-3-O-sophoroside-7-O-rhamnoside | C34H42O21 | ESI- | [M-H]- | 785.2146 | 2.61 |
|  |  | ESI+ | [M+H]^+^ | 787.2291 | 2.62 |
| Kaempferol-3-O-glucoside-7-O-rhamnoside | C27H30O15 | ESI- | [M-H]- | 593.1512 | 2.93 |
| Rutin | C27H30O16 | ESI- | [M-H]- | 609.1461 | 2.95 |
|  |  | ESI+ | [M+H]^+^ | 611.1607 | 2.96 |
| Quercetin-3-O-glucoside | C21H20O12 | ESI- | [M-H]- | 463.0882 | 3.01 |
|  |  | ESI+ | [M+H]^+^ | 465.1028 | 3.02 |
| Isorhamnetin-3-O-glucoside-7-O-rhamnoside | C28H32O16 | ESI- | [M-H]- | 623.1618 | 3.14 |
| Isorhamnetin-3-O-rutinoside | C28H32O16 | ESI- | [M-H]- | 623.1618 | 3.14 |
| Isorhamnetin-3-O-glucoside-7-O-rhamnoside | C28H32O16 | ESI+ | [M+H]^+^ | 625.1763 | 3.15 |
| Isorhamnetin-3-O-rutinoside | C28H32O16 | ESI+ | [M+H]^+^ | 625.1763 | 3.15 |
| Isorhamnetin-3-O-glucoside | C22H22O12 | ESI+ | [M+H]^+^ | 479.1184 | 3.2 |
|  |  | ESI- | [M-H]- | 477.1039 | 3.2 |
| Kaempferol-3-O-glucoside | C21H20O11 | ESI- | [M-H]- | 447.0933 | 3.3 |
| Quercetin | C15H10O7 | ESI+ | [M+H]^+^ | 303.0499 | 3.63 |
|  |  | ESI- | [M-H]- | 301.0354 | 3.63 |
| Isorhamnetin | C16H12O7 | ESI+ | [M+H]^+^ | 317.0656 | 3.92 |
|  |  | ESI- | [M-H]- | 315.051 | 3.92 |
| Kaempferol | C15H10O6 | ESI- | [M-H]- | 285.0405 | 3.95 |
| Linolenic acid (18:3) | C18H30O2 | ESI- | [M-H]- | 277.2173 | 6.42 |
| Palmitoleic acid (16:1) | C16H30O2 | ESI- | [M-H]- | 253.2173 | 6.51 |
| Linoleic acid (18:2) | C18H32O2 | ESI- | [M-H]- | 279.233 | 6.65 |
| Palmitic acid (16:0) | C16H32O2 | ESI- | [M-H]- | 255.233 | 6.86 |
| Oleic acid (18:1) | C18H34O2 | ESI- | [M-H]- | 281.2486 | 6.94 |
| Stearic acid (18:0) | C18H36O2 | ESI- | [M-H]- | 283.2643 | 7.37 |
